# Fluorescent Biosensor for Rapid and Accurate Detection of Dopamine Based on Oxidized Single-Walled Carbon Nanohorns and Cryonase Enzyme

**DOI:** 10.3390/molecules31132338

**Published:** 2026-07-03

**Authors:** Jiangnan Wang, Xiaochen Liu, Tingting Feng

**Affiliations:** College of Traditional Chinese Medicine and Food Engineering, Shanxi University of Chinese Medicine, Jinzhong 030619, China

**Keywords:** dopamine, oxidized single-walled carbon nanohorns (oxSWCNHs), aptamer, cryonase enzyme, fluorescent sensor, serum detection

## Abstract

In this study, a novel and sensitive fluorescent sensing platform was rationally developed for the rapid and specific detection of dopamine. The proposed strategy ingeniously integrates the superior adsorption behavior and extraordinary fluorescence quenching effect of oxidized single-walled carbon nanohorns toward FAM-modified aptamers, as well as the highly efficient cleavage property of the cryonase enzyme. Specifically, the fluorescently labeled aptamer is efficiently adsorbed and shielded by oxidized single-walled carbon nanohorns through strong supramolecular interactions, thereby triggering an evident fluorescence quenching response. Upon the introduction of target dopamine, the specific recognition event between dopamine and its corresponding aptamer takes place, accompanied by the formation of stable aptamer–dopamine complexes. Subsequently, these complexes are selectively hydrolyzed under the catalytic action of the cryonase enzyme, which contributes to the distinct release of fluorophores and the obvious recovery of fluorescence signals. Under the optimized experimental conditions, the aptamer exhibits good linearity toward dopamine in the concentration range of 50–400 ng/mL, with a correlation coefficient of 0.9957 and a low detection limit of 26.12 ng/mL. Therefore, the established analytical method offers a rapid, convenient, and reliable tool for the accurate determination of dopamine in complex human serum samples.

## 1. Introduction

Dopamine (DA), a neurotransmitter of profound significance, is intricately involved in multiple physiological processes, including movement control, emotion regulation, and the reward system [1,2,3]. Aberrant DA levels are associated with various neurological disorders such as Parkinson’s disease, where DA deficiency leads to motor symptoms [4,5]. Psychologically, DA is linked to motivation, pleasure, and addiction [6,7,8]. In drug development, accurate DA detection is crucial for evaluating the efficacy of medications targeting the DA system [9,10]. Thus, precise DA detection serves as a linchpin in understanding human physiology and advancing healthcare, bridging basic research and clinical practice.

Current detection techniques for DA predominantly encompass enzymatic methods [11,12], electrochemical methods [13], spectrophotometry [14], and high-performance liquid chromatography [15,16]. Nevertheless, these approaches are beset with intrinsic limitations. The operational procedures are often convoluted, the detection apparatus is costly, and the associated testing expenses are high. This complexity not only hampers the widespread implementation of these methods but also restricts their accessibility in resource-constrained settings. These inherent drawbacks thus call for the development of more straightforward, cost-effective, and efficient DA detection strategies to meet the diverse needs of various applications, from clinical diagnosis to environmental monitoring.

Aptamers, a class of oligonucleotide sequences, possess the remarkable ability to bind specifically to target molecules. These are isolated from random oligonucleotide libraries via the Systematic Evolution of Ligands by Exponential Enrichment (SELEX) technique [17,18]. Among aptamers, DNA aptamers stand out due to their outstanding biological stability, ease of labeling, and robust target-recognition capacity. As a result, they have found extensive applications in the analysis of proteins, small molecules, metal ions, and various other substances [19,20,21]. Single-walled carbon nanohorns (SWCNHs), a cutting-edge nanomaterial, exhibit remarkable efficiency as fluorescence quenchers. This unique property has been exploited in the fabrication of a plethora of optical systems. Notably, SWCNHs have been instrumental in the development of detection systems for DNA, small molecules, and macromolecules. These systems leverage the distinctive characteristics of SWCNHs, thereby underscoring their significance in the realm of optical-based detection [22,23,24]. However, the application of SWCNHs is limited in many contexts due to their hydrophobicity and chemical inertness. To address this, we subjected SWCNHs to oxidative treatment. The resulting oxidized single-walled carbon nanohorns (oxSWCNHs) exhibit improved dispersibility and enhanced chemical reactivity, rendering them more suitable for processing and application [25]. To the best of our knowledge, although a few works have used SWCNHs for DA detection via photoluminescence [26], our work is the first to integrate oxSWCNHs with aptamer and cryonase for signal amplification. Through oxidation treatment, SWCNHs are endowed with high chemical reactivity and good stability. By integrating the selective binding capability and fluorescence quenching effect of oxSWCNHs with enzyme cycling amplification using cryonase enzyme, quantitative monitoring of dopamine is achieved.

**Scheme 1:**
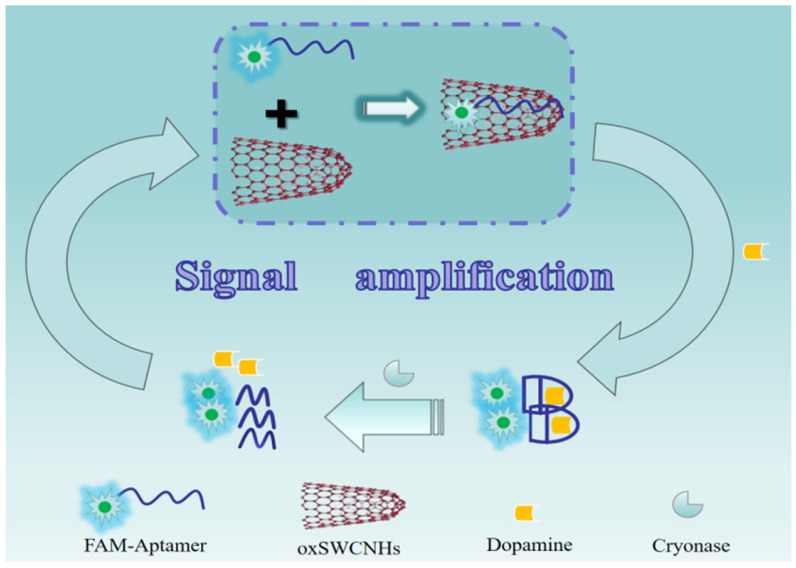
A schematic illustration of the detection mechanism of a dopamine fluorescent sensor.

Herein, we rationally engineer a highly selective fluorescent probe for the specific recognition and sensitive detection of DA by synergistically integrating three unique functional modules: the differential adsorption of oxSWCNHs toward fluorescein amidite (FAM)-labeled DA aptamers, the superior fluorescence quenching capability of oxSWCNHs, and the efficient hydrolytic activity of the cryonase enzyme. In the absence of DA, FAM-aptamer is readily adsorbed onto oxSWCNHs, where fluorescence resonance energy transfer (FRET) efficiently quenches the FAM emission; meanwhile, oxSWCNHs protect the adsorbed aptamer from cryonase enzyme -mediated cleavage. Upon introduction of DA, specific target–aptamer binding displaces the FAM-aptamer from oxSWCNHs, restoring fluorescence. The liberated DA–aptamer complex becomes susceptible to cryonase enzyme hydrolysis, releasing FAM and DA. The recycled DA then initiates successive recognition–cleavage cycles, enabling robust 1:N signal amplification and drastically boosting fluorescence output. This modular, amplification-fueled design endows the sensor with markedly enhanced selectivity and sensitivity, establishing a versatile and robust platform for DA quantification in complex biological and chemical matrices. Specifically, the probe consists of FAM-labeled DA aptamer (FAM-aptamer), oxSWCNHs, and cryonase enzyme. A schematic illustration of the DA fluorescent sensing mechanism is presented in Scheme 1.

## 2. Experimental

### 2.1. Instrumentation and Reagents

The FAM-labeled Apt (5′-FAM-GTC TCT GTG TGC GCC AGA GAA CAC TGG GGC AGA TAT GGG CCA GCA GCA CAG AAT GAG GCCC-3′) was purchased from Huzhou Hippo Biotechnology Co., Ltd. (Huzhou, China). Cryonase enzyme was obtained from Takara Biomedical Technology (Beijing) Co., Ltd. (Beijing, China). SWCNHs were purchased from Nanjing Xianfeng Nanomaterial Technology Co., Ltd. (Nanjing, China). Human serum, dopamine, NaH_2_PO_4_, phenylalanine, glutathione, methionine, arginine, proline, threonine, cysteine, L-histidine, serine, glycine, ZnSO_4_ and MgCl_2_ were obtained from Shanghai Maclin Biochemical Technology Co., Ltd. (Shanghai, China).

A pHS-3E pH meter (Shanghai Leici Co., Ltd., Shanghai, China) was used for pH measurements. Fluorescence spectra were recorded using an RF-6000 fluorescence spectrophotometer (Shimazu, Kyoto, Japan) with an excitation wavelength of 492 nm and an emission range of 510–650 nm. A PR124ZH/E electronic balance (Changzhou Ohaus Co., Ltd., Changzhou, China) was used for weighing. An MTH-100 constant-temperature mixing instrument (Hangzhou Miu Instruments Co., Ltd., Hangzhou China) was employed for incubation.

Statistical calculations and graph plotting were performed using GraphPad Prism 10 and Origin 2024 software.

### 2.2. Formulation and Fabrication of the Fluorescent Sensor

#### 2.2.1. Elaboration on the Preparation of the Buffer Solution

Precisely 0.5082 g of MgCl_2_, 0.1211 g of Tris, and 0.1463 g of NaCl were weighed. Ultrapure water was added to dissolve these components completely. Subsequently, HCl was carefully added to adjust the pH to 7.5. Finally, deionized water was introduced to bring the volume up to 500 mL, yielding a 20 mmol/L Tris-HCl solution containing 5 mmol/L MgCl_2_ and 50 mmol/L NaCl, with a pH of 7.5.

#### 2.2.2. Preparation of DA DNA Aptamer and DA Standard Solution

The DA DNA aptamer solution was formulated using ultrapure water. Initially, the aptamer (1 OD) underwent centrifugation for 1 min. Subsequently, 164 μL of ultrapure water was introduced to ensure its complete dissolution. A 100 μL aliquot of this resultant solution was then withdrawn, diluted with ultrapure water to a final volume of 2 mL. The diluted solution was carefully wrapped in tin foil and stored at −20 °C for future applications.

Precisely 5 mg of DA was weighed and dissolved in ultrapure water, bringing the volume to 5 mL. Next, a 60 μL aliquot of this solution was carefully withdrawn and further diluted with ultrapure water to a volume of 2 mL. Subsequently, 100 μL of the resulting solution was taken and diluted with water to a final volume of 2 mL. This procedure yielded a DA standard solution with a final concentration of 1.5 μg/mL. The prepared standard solution was then stored at −20 °C for subsequent use.

#### 2.2.3. Preparation of Cryonase Enzyme Solution

First, cryonase (20 U/μL) was centrifuged for 2 min. We took 25 μL of cryonase in a 2 mL EP tube then added 475 μL of DA buffer to dilute, resulting in a cryonase enzyme solution of 1 U/μL, stored at −20 °C for later use.

#### 2.2.4. Preparation of Oxidized Single-Walled Carbon Nanohorns

**Figure 1:**
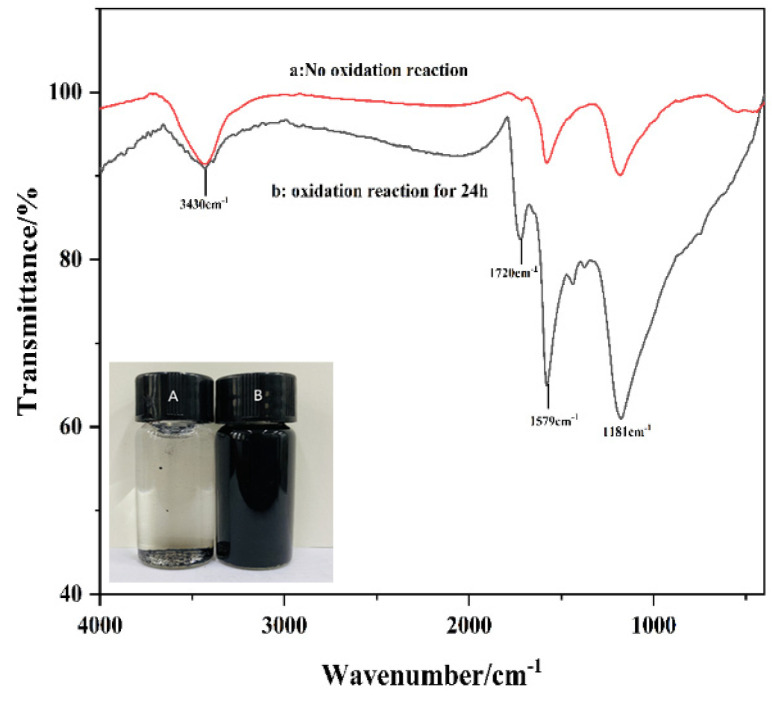
The FT−IR spectra of oxSWCNHs and SWCNHs. Inset: (**A**) dispersion of SWCNHs and (**B**) oxidized SWCNHs in aqueous solutions.

A precisely weighed 0.0040 g of SWCNH powder was placed in a 25 mL round-bottom flask, followed by the addition of 2 mL of 30% nitric acid. After allowing the mixture to stand, it was heated in an oil bath at 140 °C under reflux for 24 h. The reaction liquid was then transferred to a centrifugal tube and centrifuged. The supernatant was discarded, and ultrapure water was added. This process was repeated multiple times until the pH of the solution was close to neutral. The resulting oxSWCNHs solution was then obtained and freeze-dried for further use. The chemical groups of oxSWCNHs were characterized by Fourier transform infrared (FTIR) spectroscopy, as show in Figure 1. A strong broad peak at 3440 cm^−1^, assigned to the O-H stretching of carboxyl groups, and a peak at 1730 cm^−1^ from the C=O stretching of COOH were observed. After 24 h of oxidation, these peaks intensified, indicating increased carboxyl density on oxSWCNH surfaces. The inset in Figure 1 shows that pristine SWCNHs are insoluble in water, whereas HNO_3_-oxidized oxSWCNHs, bearing hydrophilic carboxyl groups, exhibit good water solubility.

#### 2.2.5. Preparation of Sensor

Add 15 μL of 500 nmol/L aptamer stock solution, 21 μL of 100 μg/mL oxSWCNHs solution, and 3 μL of 1 U/mL cryonase enzyme solution to 261 μL of Tris-HCl buffer and mix well. The final reaction volume will be 300 μL.

### 2.3. DA Detection Steps

Add DA standard solutions of different concentrations (50–400 ng/mL) successively to the Tris-HCl buffer containing a prepared 15 μL aptamer stock solution (500 nmol/L) and 21 μL oxSWCNHs solution (100 μg/mL), then add 3 μL cryonase enzyme solution (1 U/mL) and incubate at 37 °C for 50 min. Afterwards, measure the fluorescence spectrum using a fluorescence spectrometer (λ_ex/em_ = 492/510).

### 2.4. Assessment of DA Levels in Human Serum Samples

Under optimal conditions, the performance of the fluorescence method for determining DA was studied. Human serum was diluted 10 times, and different concentrations of DA (100, 200 and 300 ng/mL) were added to prepare serum samples. Tris-HCl buffer containing 500 nmol/L aptamer stock solution and 100 μg/mL oxSWCNHs solution was mixed with the above serum samples, then 1 U/mL cryonase enzyme solution was added, and the mixture was incubated at 37 °C for 50 min. Finally, fluorescence emission was measured and recorded in the range of 510–610 nm.

## 3. Results and Discussion

### 3.1. Feasibility Testing

**Figure 2:**
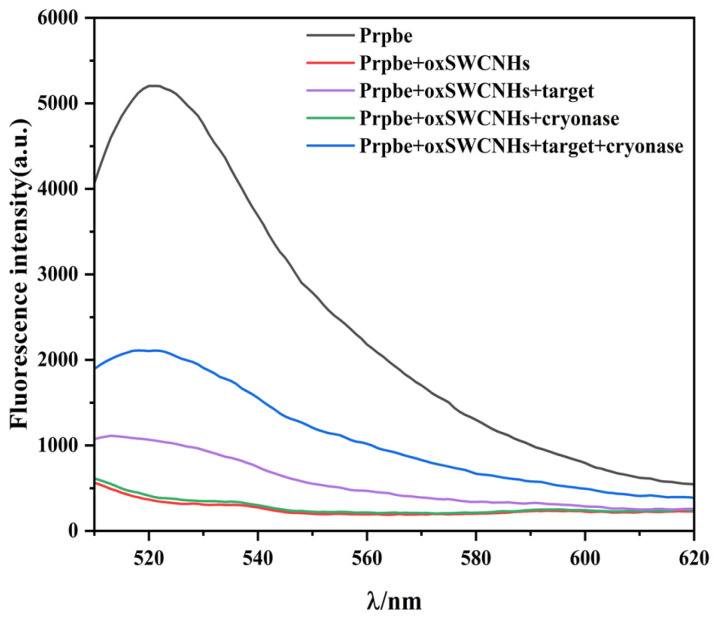
Feasibility analysis—blank line: probe (25 nmol/L); red line: probe (25 nmol/L) + oxSWCNHs (7.0 µg/mL); purple line: probe (25 nmol/L) + oxSWCNHs (7.0 µg/mL) + DA (150 ng/mL); green line: probe (25 nmol/L) + oxSWCNHs (7.0 µg/mL) + cryonase (10 mU/mL); blue line: probe (25 nmol/L) + oxSWCNHs (7.0 µg/mL) + cryonase (10 mU/mL) + DA (150 ng/mL).

The feasibility of the developed aptamer-based fluorescence sensing platform for DA detection was investigated by recording the fluorescence responses under different assay conditions, as illustrated in Figure 2. The free fluorescently labeled aptamer dispersed in buffer exhibited a strong fluorescence signal with an intensity of approximately 5300 a.u., whereas the fluorescence intensity was dramatically quenched to the minimum value after the addition of oxSWCNHs (red line), which was attributed to the strong π-π stacking interaction and high adsorption affinity between oxSWCNHs and aptamer strands that triggered efficient fluorescence quenching. Upon the subsequent introduction of DA, the fluorescence intensity was distinctly recovered (purple line) owing to the specific recognition and high-affinity binding between aptamer and DA, which induced the detachment of aptamer probes from the oxSWCNHs surface and thus weakened the quenching effect. In contrast, the fluorescence intensity showed negligible change with only cryonase enzyme added (green line), confirming that oxSWCNHs possessed robust adsorption stability and cryonase enzyme alone could not desorb the aptamer from the nanomaterial surface. Notably, a remarkably amplified fluorescence signal was achieved in the presence of both cryonase enzyme and DA (blue line), where the aptamer preferentially bound with DA to form stable complexes away from oxSWCNHs, followed by specific degradation of the aptamer–DA complexes by cryonase enzyme to release fluorescent molecules and DA, resulting in a prominent fluorescence enhancement due to the complete separation of fluorophores from the quenching interface. These sequential fluorescence variations clearly validate the rationality, working mechanism, and feasibility of the proposed sensing strategy for the sensitive and selective detection of DA.

### 3.2. Effect of oxSWCNHs Concentration and Quenching Time on the Biosensor

**Figure 3:**
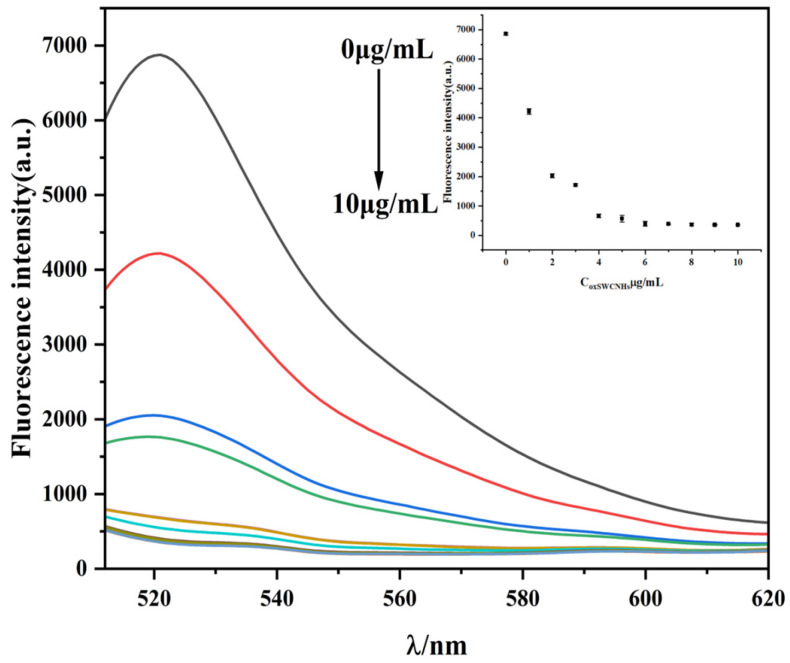
Optimization of the oxSWCNHs: (1) 0; (2) 1.0; (3) 2.0; (4) 3.0; (5) 4.0; (6) 5.0; (7) 6.0; (8) 7.0; (9) 8.0; (10) 9.0; (11) 10.0 µg/mL. Probe: 25 nmol/L.

**Figure 4:**
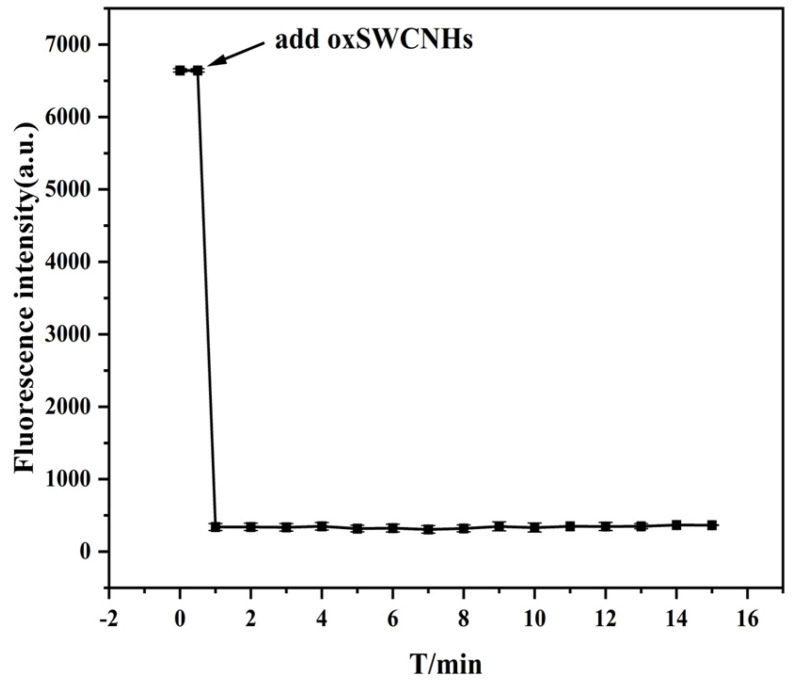
Time−dependent fluorescence intensity of the fluorescent biosensor (probe concentration: 25 nmol/L; oxSWCNHs concentration: 7 μg/mL).

To improve the sensitivity of the biosensor, optimizing the concentration and quenching time of oxSWCNHs is crucial for analytical performance. In the absence of oxSWCNHs, the fluorescence intensity of the system is relatively high, but when the concentration of oxSWCNHs increases, the fluorescence intensity gradually decreases and eventually stabilizes (Figure 3). As the concentration of oxSWCNHs reaches 7 μg/mL, the fluorescence intensity drops to less than 10% of the initial fluorescence intensity. Thus, we established 7 μg/mL as the optimal reaction concentration. As show in Figure 4, in the absence of oxSWCNHs, the fluorescence intensity of the system is relatively high, but when the oxSWCNHs (7 μg/mL) is added, the fluorescence intensity drops to its lowest level and remains stable. For experimental stability, we established 10 min as the optimal reaction time.

### 3.3. Enzyme Reaction Kinetics

**Figure 5:**
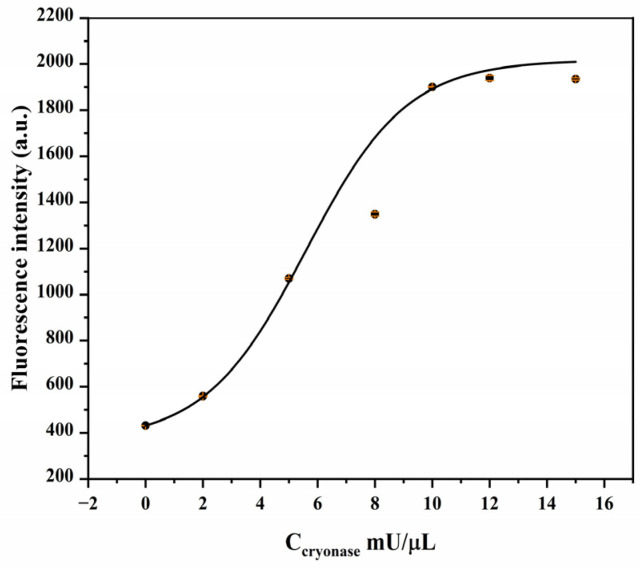
Optimization of cryonase enzyme concentration. Cryonase enzyme concentrations were set as follows: (1) 0, (2) 2, (3) 5, (4) 8, (5) 10, (6) 12, and (7) 15 mU/µL. Probe concentration was maintained at 25 nmol/L, oxSWCNHs concentration at 7 μg/mL, and DA concentration at 150 ng/mL. The excitation wavelength is 492 nm, and the maximum emission peak is at 520 nm.

**Figure 6:**
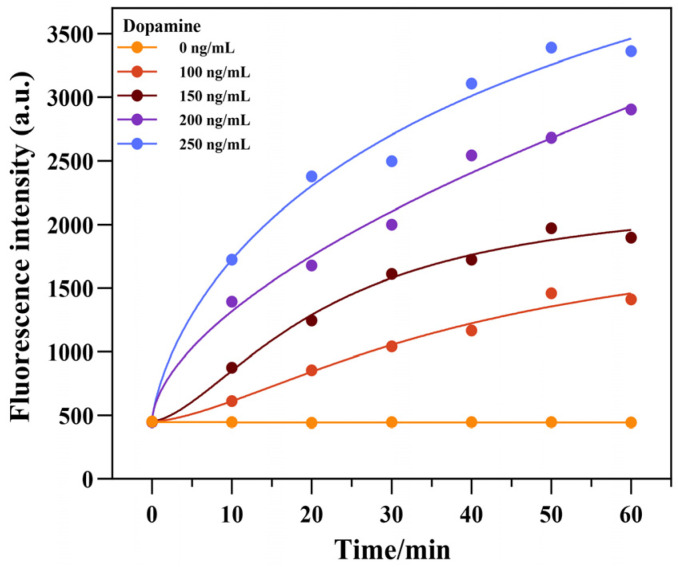
Optimization of cryonase enzyme reaction time (0, 10, 20, 30, 40, 50, 60 min). Probe concentration was fixed at 25 nmol/L, oxSWCNHs concentration at 7 μg/mL, and DA concentrations were set at 0, 100, 150, 200, and 250 ng/mL. The excitation wavelength is 492 nm, and the maximum emission peak is at 520 nm.

To investigate the effect of the cryonase enzyme on fluorescence intensity, we systematically optimized the enzyme concentration and reaction time. As depicted in Figure 5, the fluorescence intensity gradually elevated with increasing cryonase enzyme concentration and reached a plateau at 10 mU/μL. Accordingly, 10 mU/μL was adopted as the optimal concentration for subsequent experiments. Furthermore, to determine the optimal incubation period, the reaction time following cryonase enzyme addition was also evaluated. The fluorescence signals were recorded at 0, 10, 20, 30, 40, 50, and 60 min. As illustrated in Figure 6, the fluorescence intensity plateaued at 50 min, indicating that the reaction reached saturation. Therefore, 50 min was chosen as the optimal incubation time for the enzymatic reaction.

### 3.4. Quantitative Determination of DA-Related Activity

**Figure 7:**
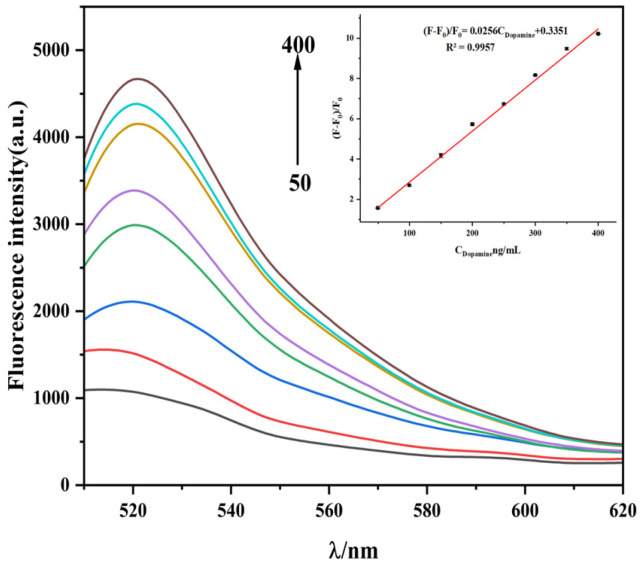
Plots of DA response within the fluorescence system. Probe concentration was maintained at 25 nmol/L, oxSWCNHs concentration at 7 μg/mL, and DA concentrations were 0, 50, 100, 150, 200, 250, 300, 350, and 400 ng/mL.

The fluorescence intensity of the system is relatively low when DA is not added. When DA is introduced into the system, the aptamer binds to DA, causing the aptamer to dissociate from the oxSWCNHs and release the fluorescent molecules, resulting in a gradual increase in the system’s fluorescence intensity, which also enhances with the increase in DA concentration. As shown in Figure 7, within the DA concentration range of 50–400 ng/mL, there is a linear relationship between the relative fluorescence intensity ratio (F − F_0_)/F_0_ of the system and the DA concentration. The regression equation is (F − F_0_)/F_0_ = 0.0256C_Dopamine_ + 0.3351 (R^2^ = 0.9957), where F represents fluorescence intensity at 520 nm in the presence of DA, and F_0_ denotes fluorescence intensity in its absence. The detection limit of this method was 26.12 ng/mL (3 S/m, where S represents the standard deviation of the reagent blank (*n* = 3) and m denotes the slope of the linear calibration curve). It indicated superior sensitivity compared to other methods (Table 1).

### 3.5. Selectivity of DA and the Impact of Potentially Interfering Substances

**Figure 8:**
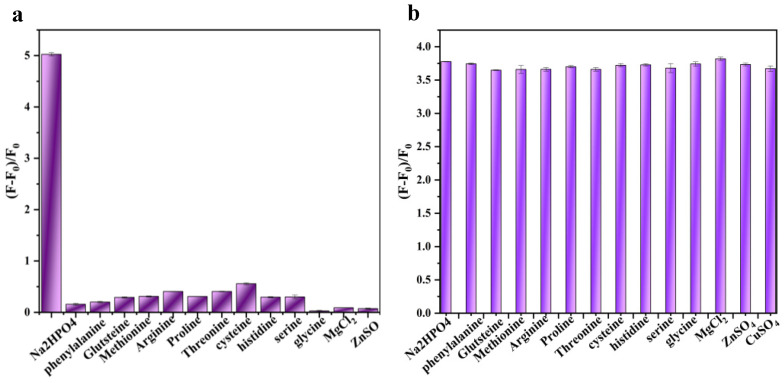
(**a**) Selectivity of the sensing system. (**b**) Interference in the sensing system (Na_2_HPO_4_, phenylalanine, glutamine, methionine, arginine, proline, threonine, cysteine, L−histidine, serine, glycine, MgCl_2_, ZnSO_4_, CuSO_4_).

The selectivity of the developed aptasensor was systematically evaluated by introducing various interfering substances, including Na_2_HPO_4_, phenylalanine, cystine, methionine, arginine, proline, threonine, cysteine, L-histidine, serine, glycine, MgCl_2_, ZnSO_4_, and CuSO_4_. As depicted in Figure 8a, negligible fluorescence responses were observed upon the addition of these interfering agents alone, indicating that they exerted no significant influence on the sensing system. Furthermore, the fluorescence responses of the aptasensor were recorded in the coexistence of DA and the above interfering substances, with the corresponding results displayed in Figure 8b. The fluorescence intensity remained almost unchanged even in the presence of these potential interferents, demonstrating that they did not interfere with the accurate determination of DA. Taken together, the results in Figure 8 convincingly confirm that the as-proposed aptasensor exhibits excellent selectivity toward DA detection.

### 3.6. Sensor Stability

**Figure 9:**
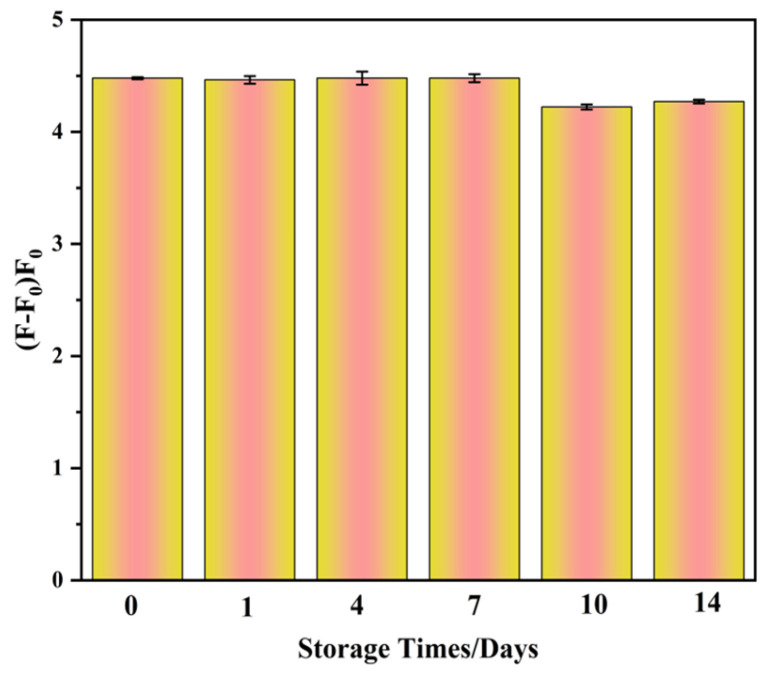
The stability of the sensor.

To investigate whether the biosensor can meet the requirements for long-term detection, its stability was systematically evaluated. Three parallel samples were prepared and stored at −20 °C to prevent degradation of FAM-labeled aptamer. The samples were placed for 0, 1, 4, 7, 10, and 14 days, respectively, and then the fluorescence intensity was measured (DA concentration of 150 ng/mL). As shown in Figure 9, the sensor can still maintain 95% of its fluorescence intensity after being placed for 14 days. This indicates that this method has good stability.

### 3.7. DA Detection in Actual Samples

To validate the feasibility of the proposed aptasensor for practical sample analysis, human serum was employed as a realistic biological matrix, taking into account that complex endogenous components may give rise to background fluorescence interference. To alleviate such interference from complicated matrices, the human serum sample was subjected to a 10-fold dilution prior to detection. As presented in Table 2, the developed sensing platform still maintained a desirable fluorescence response toward DA in the 10-fold diluted human serum. The spiked recovery experiments were further carried out to assess the quantitative performance, and the recoveries of DA were determined to be 93.24–100.74%, with relative standard deviations (RSDs) below 5%, fully satisfying the criteria for reliable quantitative detection in real biological samples.

## 4. Conclusions

In summary, a novel oxSWCNHs-based fluorescent sensing system has been successfully constructed for the sensitive detection of DA by employing oxidized single-walled carbon nanohorns as the fluorescence quencher and integrating the highly efficient hydrolysis property of the cryonase enzyme. Benefiting from the synergistic effect of the strong adsorption and protection capabilities of oxSWCNHs and the superior catalytic cleavage activity of the cryonase enzyme, the proposed sensing platform realized effective signal recycling amplification, thus significantly improving the detection sensitivity. Under optimal conditions, the developed sensor exhibited a satisfactory linear detection range from 50 to 400 ng/mL, with a low limit of detection of 26.12 ng/mL. This work not only provides a reliable and sensitive analytical strategy for DA determination but also offers a promising paradigm for the design of nanomaterial-assisted and enzyme-mediated signal amplification sensing systems, showing great potential in biomedical analysis and clinical diagnosis.

## Figures and Tables

**Table 1:** Comparison of different methods for detecting DA.

Analytical Method	Material	Detection Range (ng/mL)	LOD (ng/mL)	Reference
Spectrophotometry	2,4-Dinitrophenylhydrazine	5000–50,000	320	[14]
HPLC	Acetate Buffer and Methanol	-	30	[16]
Fluorescence	SWCNHs	3063.6–336,996	765.9	[26]
Electrochemistry	Graphene	612.72–15,318	398.27	[27]
Electrochemistry	Reduced graphene oxide	153.18–12,254.4	70.46	[28]
Fluorescence	Silver-based Peroxidase-Mimetic Nanozymes	76.59–12,254.4	35.23	[29]
Fluorescence	MoS_2_ Quantum Dots	30,636–91,908	612.7	[30]
Fluorescence	oxSWCNHs	50–400	26.12	This work

**Table 2:** Recovery of DA in human serum.

Sample	Detected (ng/mL)	Added (ng/mL)	Found (ng/mL)	Recovery (%)	RSD (%, *n* = 3)
1	0.026	0	-	-	-
2	0.029	100	97.25	97.25	1.13
3	0.038	200	201.48	100.74	0.48
4	0.034	300	279.78	93.24	0.12

## Data Availability

The original contributions presented in this study are included in the article. Further inquiries can be directed to the corresponding author.

## References

[1] Li P., Ma Q., Zhu F., Song K., Liu Y., Jiang Y., Mao L. (2025). In vivo electrochemical aptasensors reveal reversible dopamine dysregulation from adolescent malnutrition. ACS Sens..

[2] Liu Y., Jin Y., Zhu F., Li X., Ma C., Sun Z., Yao Y., Song K., Mao L., Jiang Y. (2026). Mirror-image L-DNA aptamers enable stable in vivo dopamine sensing. J. Am. Chem. Soc..

[3] Zheng Y., Cai R., Wang K., Zhang J., Zhuo Y., Dong H., Zhang Y., Wang Y., Deng F., Ji E. (2025). In vivo multiplex imaging of dynamic neurochemical networks with designed far-red dopamine sensors. Science.

[4] Mkrtchian A., Qiu Z.G., Abir Y., Erdmann T., Dercon Q., Sedlinska T., Browning M., Costello H., Huys Q.J.M. (2025). Differential associations of dopamine and serotonin with reward and punishment processes in humans: A systematic review and meta-analysis. JAMA Psychiatry.

[5] Roy T., Banerjee R., Chatterjee A., Swarnakar S. (2025). Dopamine toxicity induces ROS-dependent death of murine neuroblastoma cells: Impact on the interactions of cofilin with UCHL1 and MMP9. Neurochem. Res..

[6] Mansour S., Abdelrahman A., Banwari G. (2025). Dopamine dysfunction beyond psychosis: Reevaluating its role in depression, anxiety, and obsessive-compulsive disorder. Psychiatr. Danub..

[7] Yin T.S., Zhao Y.Q., Zhang J.Q., Xiao X., Huang Y., Ke B.L., Huang Z.J. (2025). Ultrasensitive and selective detection of dopamine through substituent-regulated evolution of quantum defects. ACS Sens..

[8] Yang Y.J., Li X.Y., Lu J.Y., Ge J.J., Chen M.J., Yao R.X., Tian M., Wang J., Liu F.T., Zuo C.T. (2025). Recent progress in the applications of presynaptic dopaminergic positron emission tomography imaging in parkinsonism. Neural Regen. Res..

[9] Ardila Jurado E., Zünd-Hofer L., Brugger F., Nicastro N., Bhatia K.P., Kägi G. (2025). The role of presynaptic dopaminergic imaging in acquired neurological conditions affecting basal ganglia: A systematic review. Front. Neurol..

[10] Moreau C., Odou P., Labreuche J., Demailly A., Touzet G., Reyns N., Gouges B., Duhamel A., Barthelemy C., Lannoy D. (2025). Intracerebroventricular anaerobic dopamine in parkinson’s disease with L-dopa-related complications: A phase 1/2 randomized-controlled trial. Nat. Med..

[11] Beatto T.G., Gomes W.E., Etchegaray A., Gupta R. (2023). Dopamine levels determined in synthetic urine using an electrochemical tyrosinase biosensor based on ZnO@Au core-shell. RSC Adv..

[12] Ravariu C. (2023). From enzymatic dopamine biosensors to OECT biosensors of dopamine. Biosensors.

[13] Lakard S., Pavel I.-A., Lakard B. (2021). Electrochemical Biosensing of Dopamine Neurotransmitter: A Review. Biosensors.

[14] Ramadan M., Almasri I., Khayal G. (2020). Spectrophotometric determination of dopamine in bulk and dosage forms using 2,4-dinitrophenylhydrazine. Turk. J. Pharm. Sci..

[15] Guiard B.P., Gotti G. (2024). The high-precision liquid chromatography with electrochemical detection (HPLC-ECD) for monoamines neurotransmitters and their metabolites: A review. Molecules.

[16] Baranowska I., Joanna Płonka J. (2008). Determination of levodopa and biogenic amines in urine samples using high-performance liquid chromatography. J. Chromatogr. Sci..

[17] Ellington A.D., Szostak J.W. (1990). In vitro selection of RNA molecules that bind specific ligands. Nature.

[18] Tuerk C., Gold L. (1990). Systematic evolution of ligands by exponential enrichment: RNA ligands to bacteriophage T4 DNA polymerase. Science.

[19] Jayasena S.D. (1999). Aptamers: An emerging class of molecules that rival antibodies in diagnostics. Clin. Chem..

[20] Mosing R.K., Bowser M.T. (2009). Isolating aptamers using capillary electrophoresis-SELEX (CE-SELEX). Methods Mol. Biol..

[21] Xiang D., Shigdar S., Qiao G., Wang T., Kouzani A.Z., Zhou S.F., Kong L., Li Y., Pu C., Duan W. (2015). Nucleic acid aptamer-guided cancer therapeutics and diagnostics: The next generation of cancer medicine. Theranostics.

[22] Feng T.T., Yan S.Z., Huang Y. (2024). Novel Enzyme-assisted recycle amplification strategy for tetracycline detection based on oxidized single-walled carbon nanohorns. Molecules.

[23] Zieba W., Olejnik P., Koter S., Kowalczyk P., Plonska-Brzezinska M.E., Terzyk A.P. (2020). Opening the internal structure for transport of ions: Improvement of the structural and chemical properties of single-walled carbon nanohorns for supercapacitor electrodes. RSC Adv..

[24] Zhu S.Y., Xu G.B. (2010). Single-walled carbon nanohorns and their applications. Nanoscale.

[25] Liu X., Ying Y., Ping J. (2020). Structure, synthesis, and sensing applications of single-walled carbon nanohorns. Biosens. Bioelec-Tronics.

[26] Zhang J., Hou S., Zhang J., Liang N., Zhao L. (2020). A facile aptamer-based sensing strategy for dopamine detection through the fluores-cence energy transfer between dye and single-wall carbon nanohorns. Spectrochim. Acta. Part A Mol. Biomol. Spectrosc..

[27] Kim Y.R., Bong S., Kang Y.J., Yang Y., Mahajan R.K., Kim J.S., Kim H. (2010). Electrochemical detection of dopamine in the presence of ascorbic acid using graphene modified electrodes. Biosens. Bioelectron..

[28] Yu J., Kim T.H. (2017). A facile electrochemical fabrication of reduced graphene oxide-modified glassy carbon electrode for simultaneous detection of dopamine, ascorbic acid, and uric acid. J. Electrochem. Sci. Technol..

[29] Chen Y., Zhou C., Zhang W., Wang H., Su X. (2026). Bifunctional fluorescent silver-based peroxidase-mimetic nanozymes: A novel ratiometric fluorescence/colorimetric dual-signal system for dopamine detection. Talanta.

[30] Zhao Y., Yang F., Zhao L., Xu G. (2025). A simple and rapid fluorescent sensor based on MoS_2_ quantum dots for dopamine detection. J. Fluoresc..

